# The Safe and Effective Use of Plant-Based Diets with Guidelines for Health Professionals

**DOI:** 10.3390/nu13114144

**Published:** 2021-11-19

**Authors:** Winston J. Craig, Ann Reed Mangels, Ujué Fresán, Kate Marsh, Fayth L. Miles, Angela V. Saunders, Ella H. Haddad, Celine E. Heskey, Patricia Johnston, Enette Larson-Meyer, Michael Orlich

**Affiliations:** 1 Center for Nutrition, Healthy Lifestyles, and Disease Prevention, School of Public Health, Loma Linda University, Loma Linda, CA 92354, USA; fmiles@llu.edu (F.L.M.); ehaddad@llu.edu (E.H.H.); cheskey@llu.edu (C.E.H.); pjohnston@llu.edu (P.J.); 2 Vegetarian Resource Group, Baltimore, MD 21203, USA; reedmangela@gmail.com; 3 eHealth Group, Instituto de Salud Global Barcelona (ISGlobal), 08036 Barcelona, Spain; ujue.fresan@isglobal.org; 4 Private Practice, Chatswood, NSW 2067, Australia; drkatemarsh@gmail.com; 5 School of Medicine, Loma Linda University, Loma Linda, CA 92354, USA; morlich@llu.edu; 6 Nutrition Insights, Sanitarium Health Food Company, Berkeley Vale, NSW 2261, Australia; angela.saunders@sanitarium.com.au; 7 Human Nutrition, Foods, and Exercise Virginia Tech, Blacksburg, VA 24061, USA; enette@vt.edu

**Keywords:** plant-based diets, vegetarian, vegan, sustainability, microbiome, vitamin B12, CV disease, diabetes, bone health, life cycle

## Abstract

Plant-based diets, defined here as including both vegan and lacto-ovo-vegetarian diets, are growing in popularity throughout the Western world for various reasons, including concerns for human health and the health of the planet. Plant-based diets are more environmentally sustainable than meat-based diets and have a reduced environmental impact, including producing lower levels of greenhouse gas emissions. Dietary guidelines are normally formulated to enhance the health of society, reduce the risk of chronic diseases, and prevent nutritional deficiencies. We reviewed the scientific data on plant-based diets to summarize their preventative and therapeutic role in cardiovascular disease, cancer, diabetes, obesity, and osteoporosis. Consuming plant-based diets is safe and effective for all stages of the life cycle, from pregnancy and lactation, to childhood, to old age. Plant-based diets, which are high in fiber and polyphenolics, are also associated with a diverse gut microbiota, producing metabolites that have anti-inflammatory functions that may help manage disease processes. Concerns about the adequate intake of a number of nutrients, including vitamin B12, calcium, vitamin D, iron, zinc, and omega-3 fats, are discussed. The use of fortified foods and/or supplements as well as appropriate food choices are outlined for each nutrient. Finally, guidelines are suggested for health professionals working with clients consuming plant-based diets.

## 1. Introduction

Interest in plant-based diets has soared in the past decade for a myriad of reasons [1]. People are concerned about issues such as their health, climate change, the sustainability of the food production system, and the welfare of animals. A plant-based diet is defined in various ways. For some it means eating foods mostly, but not entirely, of plant origin, while for others it means eating only plant-based foods. In this manuscript, we chose to restrict the term plant-based to include both vegan diets (total plant-based nutrition) and lacto-ovo-vegetarian diets (this allows for the consumption of dairy products and eggs). We do not include a discussion of flexitarian, semi-vegetarian, or pesco-vegetarian eating patterns, as they do not fit into our definition of plant-based diets.

This paper will discuss the environmental issues and the benefits for the planet of significantly reducing or eliminating meat and dairy foods from our diet. In addition, it outlines the therapeutic advantages of a plant-based diet for managing the chronic diseases of Western society, such as obesity, cardiovascular disease (CVD), cancer, and diabetes. A plant-based diet is also shown to have a substantial impact upon the composition and function of the gut microbiome, which in turn influences our overall health. Furthermore, the safety of following a plant-based diet during all stages of the life-cycle is addressed. Finally, questions were raised about the adequacy of a plant-based diet with respect to eight key nutrients. These are discussed in detail, with solutions suggested as to how one can meet the dietary requirements through food choices and/or supplementation. Some simple guidelines are given for health professionals to effectively serve the growing population of those consuming a plant-based diet.

## 2. Current Trends

Internationally, the prevalence of following a vegetarian diet varies by country, but it is generally estimated to be less than 10% of the population. The exception is India, where 20% or more of adults are vegetarian [2,3]. In the United States, a nationwide poll in 2020 found that approximately 6% of adults followed a vegetarian diet, with half of them being vegans [4]. A similar U.S. poll found that approximately 2% of 8- to 17-year-old children followed a vegan diet, and 3% followed a non-vegan vegetarian diet [5].

Globally, the market for alternatives to dairy products is expected to reach $US 25 billion by 2026 [6]. U.S. retail sales of plant-based foods (plant-based dairy alternatives and plant-based meats) increased 27% between 2019 and 2020, with a total plant-based market value estimated at $7 billion [7], suggesting a growing consumer interest in non-animal products.

The 2020–2025 Dietary Guidelines for Americans endorses a “Healthy Vegetarian Dietary Pattern” as one of three dietary patterns that can “be tailored to meet cultural and personal preferences” [8]. There are versions of this plan for ages one year and older. The Guidelines also encourage all Americans to eat more plant foods, including dried beans, whole grains, fruits, vegetables, and nuts. Many other countries promote plant-based diets in their dietary guidelines [9].

With a growing interest in vegetarian eating, establishments such as colleges and universities, school food services, airlines, restaurants, prisons, employee food services, nursing homes, and hospitals are increasingly providing vegetarian options [10,11,12].

## 3. Environmental Sustainability of Vegetarian Diets

The production of different foods can have very diverse environmental impacts. There is a large variation in the ecological footprint of animal-based products, with ruminant meat being especially detrimental for the environment as compared with other products such as pork, white meat, or eggs [13,14,15]. An increasing body of data provides evidence that environmental degradation, through the emissions of greenhouse gas (GHG) and other pollutants, and the use of earth’s resources, such as water and land, in the production of plant-based foods are significantly lower than that from animal-based foods [13,14,15]. Certainly, the effects of the lowest-impact animal products are typically greater than those of plant-based alternatives, even in the case of highly processed plant-based meat analogs [14,16]. The production of plant-based products is more efficient regardless of whether the comparison is made by weight of product, per serving, per calories, or even protein content [14,15,17,18]. Producing the same amount of protein from tofu (soybeans) in comparison to beef protein requires 74 times less land and eight times less water, while the GHG emissions are 25 times lower and the eutrophication (a process driven by the enrichment of water by nutrients, especially compounds of nitrogen and/or phosphorus, leading to an increased growth, primary production and biomass of algae; changes in the balance of organisms; and water quality degradation [19]) potential is reduced by 39 times [14,18]. Even if compared to egg protein, tofu protein requires almost three times less land and six times less water, while the GHG emissions are only half of that from egg protein, and the eutrophication potential is five times lower [14,18].

Likewise, a reduction of animal-based foods in the diet goes hand in hand with a decrease in the dietary environmental impact [20,21]. Vegetarian diets, both lacto-ovo-vegetarian and vegan, have been described as more environmentally sustainable than those diets including meat. A review study concluded that the adoption of lacto-ovo-vegetarian diets could reduce the dietary GHG emissions by 35%, land use by 42%, and freshwater use by 28% [20]. Adopting a vegan diet would lead to around one-half of both GHG emissions and land use of that of current dietary patterns. One should note that there is substantial variability in the dietary environmental impact of those consuming vegetarian diets. In the final analysis, any environmental benefits would depend on the quantity and the specific foods consumed. Overconsumption of calories, a high intake of fruits transported by plane, or the consumption of large quantities of fatty dairy products, such as cheese or butter, in lacto-ovo-vegetarian diets could jeopardize any potential benefit from the avoidance of meat.

Studies suggest that the adoption of nutritionally balanced vegetarian diets, both in developed and developing countries, could be an effective strategy for reducing GHG emissions worldwide [22,23]. This dietary transition would be moderately effective in reducing fertilizer application and would decrease, although to a lesser extent, cropland and fresh water use [23]. Altogether, embracing a balanced vegetarian diet, especially in developed countries, could be an effective strategy for reducing the food system’s environmental degradation and reducing our use of the earth’s resources.

## 4. Plant-Based Diets and Chronic Diseases

### 4.1. CVD, including Hyperlipidemia, Ischemic Heart Disease, Hypertension, and Stroke

CVD continues as the most common cause of death and disability in the U.S. and globally [24,25]. The leading risk factors for CVD include dyslipidemia, excess weight, hypertension, glucometabolic disorders, and diabetes and are attributed to poor diets [26,27]. Compared to omnivorous diets, vegetarian and plant-based diets rich in whole grains, legumes, vegetables, fruits, nuts, and seeds have been associated with substantial reductions in several modifiable risk factors, including body mass index (BMI) and waist circumference [28,29], atherogenic lipoprotein concentrations [29,30], blood glucose [28], inflammation [31], and blood pressure [32].

The results of randomized controlled trials (RCT) of vegan and vegetarian interventions along with systematic reviews and meta-analyses of such studies show improvements in several intermediate cardiometabolic risk markers, including body weight and blood lipids [33,34,35,36,37], and cardiometabolic risk profiles [38]. Data from relatively long-term (years) clinical intervention studies with intensive low-fat vegetarian [39] and vegan diets [40] show reversals in coronary artery disease in individuals with CVD. Due to lower saturated fat and cholesterol levels and more optimal plant sterol and fiber content, greater favorable effects of vegan diets on heart disease risk factors are expected. A vegan diet, compared to the American Heart Association (Dallas, TX, USA) diet for coronary heart disease (CHD), resulted in similar reductions in BMI, waist circumference, markers of glycemic control, blood lipids, and a 32% lower high-sensitivity C-reactive protein (a pro-inflammatory marker) [31].

In the Adventist Health Study-2 (AHS-2), vegetarians had a 13% and 19% lower risk of CVD and ischemic heart disease (IHD) mortality, respectively, compared with non-vegetarians. This difference occurred in spite of the fact that the non-vegetarians in the cohort consumed less meat than the general population. Blood pressure levels in vegans and vegetarians were also lower than those of the omnivores. Metabolic syndrome and type 2 diabetes (T2D) are prime risk conditions for CVD and stroke. A reduced prevalence of these conditions was observed in vegan and vegetarian participants of AHS-2 [28,41].

The EPIC-Oxford study of vegetarians, vegans, and health-conscious individuals reported that the risk of incident IHD hospitalizations and deaths caused by circulatory disease was 32% lower in vegetarians than in non-vegetarians [42]. The 18-year follow-up showed lower rates of IHD in vegetarians but higher rates of hemorrhagic and total stroke [43]. Red meat intake, both processed and unprocessed, was associated with CHD risk in male health professionals [44]. In the large prospective cohort of men and women of the US National Institutes of Health—AARP Diet and Health Study, higher plant protein intake was associated with reduced CVD mortality [45]. In the Atherosclerosis Risk in Communities study, higher intakes of plant-based diets scored as healthy were associated with a lower risk of incident CVD and CVD mortality [46].

Pooled data from seven prospective cohort studies showed a reduced CHD incidence and mortality of 28% and 22%, respectively, associated with vegetarian diets. No association with CVD or stroke mortality was seen [47]. Similarly, a comprehensive review and meta-analysis of 10 prospective cohort studies showed a 25% reduced risk of incidence or mortality from IHD in vegetarian and vegan diets but not of total CVD and stroke mortality [48]. CVD and stroke mortality outcomes may be influenced by lifestyle factors other than diet and by access to cardiovascular healthcare.

### 4.2. Type 2 Diabetes

Observational studies in a variety of populations have consistently shown that compared to non-vegetarians, those following a vegetarian or vegan diet have a significantly lower risk of T2D [41,49,50,51,52,53]. A 2017 systematic review and meta-analysis of 14 studies found a pooled odds ratio for diabetes in vegetarians vs. non-vegetarians of 0.73 [54]. A 2020 systematic review similarly found that a vegan diet was associated with lower prevalence or incidence of T2D, although in some studies it was not possible to determine if the benefits were due to the vegan diet alone or combined with other healthy lifestyle habits [55].

A 2018 systematic review of nine RCTs found that, compared to control diets (including those of several diabetes associations), plant-based diets were associated with significant improvement in emotional well-being, physical well-being, depression, quality of life, general health, HbA1c levels (a measure of long-term blood glucose levels), weight, and total and LDL cholesterol levels [56]. An earlier systematic review and meta-analysis of six studies found that the consumption of vegetarian diets was associated with a significant reduction in HbA1c compared to control diets [57]. Similarly, a reduction in HbA1c has been observed with plant-based diets, including vegetarian, vegan, Mediterranean, and Dietary Approaches to Stop Hypertension (DASH) diets, compared to control or conventional diets [58].

There are several possible explanations for the benefits of plant-based diets for diabetes prevention and management. Compared to most western diets, vegetarian and vegan diets are generally higher in dietary fiber and are likely to include more whole grains, legumes and nuts, all of which have been associated with a reduced risk of T2D [59]. There is also evidence for an inverse association between higher intakes of green leafy vegetables and fruit and the risk of T2D [60,61,62,63].

The absence or limited intake of animal protein and red meat also likely plays a role. At least 25 studies have been published assessing the relationship between meat intake and T2D risk, with the majority showing a positive association between red meat and/or processed meat intakes. A 2013 meta-analysis found an association between higher intakes of total meat, unprocessed red meat, and processed meat and T2D risk [64]. There is also consistent evidence for an association between total dietary heme iron intake and heme iron intake from red meat and risk of T2D, and high serum ferritin levels are associated with insulin resistance and T2D risk [65]. A 2019 meta-analysis of prospective cohort studies looking at dietary protein intake and subsequent risk of T2D found high total protein and animal protein intakes to be associated with an increased risk of T2D, while a moderate plant protein intake was associated with a decreased risk [66]. An earlier systematic review and meta-analysis of 13 RCTs in people with diabetes found that replacing animal protein with plant protein (around 35% of total protein/day) resulted in significant reductions in HbA1c, fasting glucose, and fasting insulin levels compared to control groups [67].

Excess weight is a significant contributor to insulin resistance and T2D risk, and weight loss is a key component of the management of T2D [68]. Following a vegetarian or vegan diet, one is less likely to be overweight [69].

### 4.3. Cancer

Each of the plant food-groups has shown that they possess chemo-protective properties. Systematic reviews and meta-analyses have shown that an increased nut consumption was associated with both a decreased risk of all cancers combined [70] and decreased cancer mortality [71]. In the same manner, an increased intake of fruits and vegetables and of whole grains was shown to decrease the risk of total cancer incidence [72] and total cancer mortality [73], respectively. Furthermore, a higher intake of legumes (beans and lentils) was associated with a decrease in the risk of gastro-intestinal cancers and all cancer sites combined [74]. Many plant foods are rich in health-promoting phytochemicals, some of which have been shown to be useful in the treatment of human cancer [75,76].

On the other hand, the consumption of 100–120 g/day of red meat significantly increased the risk of many cancers (compared to eating no meat): 11% for breast cancer, 17% for colorectal cancer, and 19% for advanced prostate cancer [77]. For the consumption of 50 g/day of processed meat, the risk was increased 4% for total prostate cancer, 9% for breast cancer, 18% for colorectal cancer, 19% for pancreatic cancer, and 8% for cancer mortality [77]. In the French NutriNet-Santé cohort study, red meat intake was associated with increased risk of overall cancers (HR 1.31) and breast cancer (HR 1.83), but not prostate cancer [78]. In the National Institutes of Health (Rockville, MD, USA)—AARP Diet and Health Study cohort of half a million people, aged 50 to 71 years at baseline, higher red and processed meat intakes were associated with modest increases in total and cancer mortality [79].

With the elimination of meat and a greater use of protective plant foods, vegetarians may have a reduced risk of cancer. Epidemiologic cohort studies in the U.S. and UK have provided high-quality evidence regarding the association of vegetarian dietary patterns with cancer risk. In the US-based AHS–2, vegans had lower overall cancer risk compared to non-vegetarians (HR 0.84); overall cancer risk for lacto-ovo vegetarians was not significantly different from non-vegetarians [80]. Vegans showed a lower risk of prostate cancer (HR 0.65) [81] and a lower (but not statistically significantly lower) risk of breast cancer [82]. Neither lacto-ovo-vegetarians or vegans had a significantly lower risk of colorectal cancer [83].

In the UK-based EPIC-Oxford study, compared with meat eaters, vegans (HR 0.82) and lacto-ovo vegetarians (HR 0.90) had lower risk of all cancers combined [84]. For prostate cancer, while vegans (HR 0.61) and vegetarians (HR 0.86) had lower risk, they were not significantly different from meat eaters [84]. For colorectal cancer and female breast cancer, risk for the vegetarian groups again did not significantly differ from meat eaters [84]. In the UK Women’s Cohort Study, compared with red meat eaters, the risk of breast cancer for vegetarians was not significantly lower (HR 0.85) [85].

Taken as a whole, such results seem to support the idea that vegetarians (including vegans and lacto-ovo vegetarians) have a modest but potentially important reduced overall cancer risk compared to their non-vegetarian counterparts. Findings for common individual cancers (colorectal, prostate, breast) are less consistent and warrant further study.

### 4.4. Overweight and Obesity

Over 70% of adults in the U.S. are overweight or obese [86], and trends show that overweight and obesity are increasing worldwide [87]. Observational studies show that vegans and vegetarians typically have a lower BMI than omnivores [88,89], and vegetarian diets or plant-based type dietary patterns are protective against adult weight gain and/or the risk of overweight or obesity [90,91]. Vegans typically have the lowest BMI or lowest prevalence of overweight or obesity in studies that compare multiple dietary patterns, including vegetarians and omnivores [88,92]. Gogga et al. noted differences in percent body fat between vegans, lacto-ovo-vegetarians, and omnivores, even though all group BMI values were within the normal range [93]. Interventions using vegan [94,95,96,97,98,99], vegetarian [97,100], or whole-food plant-based dietary [101] treatments have been found to lower BMI, weight, or fat-mass compared to subjects on a meat-based diet. A 4.8% weight loss was reported for overweight and obese subjects randomized to a vegan or vegetarian diet for 2 months, compared to a 2.2% loss seen in those consuming an omnivorous diet [97]. Weight loss of 3 to 5% is clinically meaningful and may contribute to chronic disease risk factor reduction [102].

The quality of the plant-based diet is also an important consideration. Subjects who adhere to a healthy plant-based diet are reported to have a lower BMI, waist circumference, and visceral fat than those who adhere to ‘unhealthy’ plant-based diets [103,104]. Researchers have noted that diet quality may be more important than dietary patterns when comparing vegans, vegetarians, and omnivores, as the adiposity values did not differ significantly between these groups [105]. The weight loss experienced on a hypocaloric lacto-ovo-vegetarian diet was similar to that observed with a hypocaloric Mediterranean diet [106].

Mechanisms that explain the weight management benefits of plant-based diets include relatively higher fiber, fruit, and vegetable consumption compared to omnivorous diets [88,107]. This food pattern may lead to beneficial alterations to appetite hormones [93,108] and the gut microbiota [109], both of which may have an impact on body weight.

### 4.5. Bone Health

Healthy bones require a variety of essential nutrients and healthy lifestyle practices to maximize peak bone mass during growth and minimize bone loss later in life [110]. While calcium and vitamin D are well recognized as important contributors to bone health, other nutrients, including magnesium, potassium, vitamin K, vitamin C, and zinc, as well as bioactive compounds found in fruits and vegetables, have been suggested as contributing to bone health and/or reduced risk of fracture [111,112,113,114]. Some have reported greater benefit from vegetables, especially cruciferous and allium vegetables, than from fruit [115,116].

The relationship of protein intake to bone status is complex. Earlier studies showed high intakes cause a loss of calcium, while a recent review found “no adverse effects of higher protein intakes” and some positive trends at most bone sites [117]. A recent review and meta-analysis found no difference between soy and animal protein on bone mineral density (BMD) and certain markers of bone turnover [118]. Others suggest the low acid load of vegetarian diets, partly due to the potassium and magnesium content from an increased fruit and vegetable intake, is beneficial to bone health [119]. Some elderly vegetarians and a few vegans may not consume sufficient protein for maintaining optimal bone health [114,120,121].

The impact of a vegetarian diet on bone health has many dimensions. Reports can vary considerably in study design, populations, and conclusions. Some find significantly lower BMD in vegetarians, especially vegans, which could increase fracture risk [122], while others see no difference in bone health, provided that calcium and vitamin D is adequate [123], and conclude that vegetarian food can provide a solid foundation for healthy bones and preventing fractures [124].

A large prospective UK study found that fish eaters and vegetarians had a higher risk of hip fractures compared to meat eaters, while vegans had a greater risk of total, hip, leg, and vertebral fractures [125]. Some of the differences may have been partly due to lower BMI and possibly lower intake of calcium and protein in the vegans.

A systematic review of some 20 studies involving 37,134 subjects found vegetarians and vegans had lower BMD at the femoral neck and lumbar spine compared to omnivores [126]. The effect was greater in vegans who also had higher fracture rates [127,128]. Another review concluded that the balance between protective factors in vegetarian and vegan diets and potential nutrient shortfalls may leave vegetarians, and especially vegans, at increased risk of bone loss and fractures [129]. Potential nutrient shortfalls can be remedied by appropriate food selections (including fortified foods) containing critical nutrients or by taking supplements. More research data on the bone health of vegans are needed before definitive recommendations can be made.

## 5. Eating Disorders

Previous use of a vegetarian or vegan diet apparently does not increase the risk of developing any eating disorder, such as anorexia nervosa, bulimia nervosa, and binge eating disorder [130,131]. Those with preexisting disordered eating tendencies may select vegetarian or partially vegetarian diets as a way to limit food intake in a socially acceptable fashion [130,132]. Semi-vegetarians appear to be at higher risk for developing eating disorders than vegetarians and vegans [130,133]. Those vegetarians whose motivation is weight control report more symptoms of disordered eating than do those with other motivations [134]. Commonly used assessment tools may incorrectly assess dietary restraint or eating disorder psychopathology in vegetarians [130].

## 6. Plant-Based Diets and the Gut Microbiome

The human gut microbiota is a highly complex community of some 10^14^ microorganisms. Diet has a significant impact upon the microbiota composition and function [135,136]. The microbiome has a profound impact on one’s personal health and wellbeing [137]. Manipulating the gut microbiota has been viewed as a way to modulate the risk of chronic diseases such as obesity, T2D, cancer, and CVD [135,137].

Gut microbiota have a major role in the fermentation of nondigestible carbohydrates, namely resistant starch, soluble/insoluble dietary fiber, including plant wall polysaccharides and oligosaccharides. Fermentation of these nondigestible carbohydrates is associated with a higher abundance of microbes that produce butyrate and other short-chain fatty acids, which have an anti-inflammatory function, strengthen the intestinal barrier function, and improve overall gut health [138,139,140,141]. For example, the consumption of fiber-rich foods such as barley, wheat bran, brown rice, and other whole grains, as well as fructo-oligosaccharides and other prebiotics, are reported to increase butyrate-producing microbes [137,142,143,144,145,146]. Vegetarians would be expected to have an increased abundance of these microbes, as their fiber-rich diets are typically high in whole grains, fruits, vegetables, nuts, and legumes [107].

These plant foods also contain polyphenols—lignans, isoflavones, anthocyanins, and flavonols—in addition to other phytochemicals such as carotenoids and phytosterols [147,148,149]. These are metabolized into bioactive compounds by various microbes [150], some with health benefits and anti-inflammatory or antioxidant activity. Phytochemicals increase beneficial bacteria, including *Lactobacillus* and *Bifidobacterium*, which are the primary species present in probiotic supplements that are taken to improve gut health [151], in addition to some butyrate producers [150]. Among fiber-rich plant foods, nuts in particular (walnuts, almonds, pistachios) have been found to have prebiotic effects and are associated with increases in butyrate-producing microbes and other beneficial microbes [152]. Hence, the gut microbial composition is greatly influenced by dietary fiber as well as by polyphenols and other phytochemicals and their metabolites, all of which are more highly consumed by vegetarians.

Studies have supported the value of two so-called enterotypes, or clusters of microbes driven by distinct genera, in distinguishing dietary patterns. Accordingly, Bacteroides are associated with animal fat and high-protein diets [153,154,155,156,157], and *Prevotella* are associated with fiber-rich foods and carbohydrates, typical of a plant-based diet [158,159,160]. Higher abundance of *Prevotella* and other polysaccharide-degrading or potential butyrate-producing microbes has been seen particularly in agrarian cultures such as those in Tanzania, the Peruvian Amazon, and Burkina Faso, compared to U.S. or Western populations, reflecting the higher consumption of fiber-rich plant foods by these societies [160,161,162]. Hence, enterotypes may have some utility in distinguishing plant- and animal-based diets. Plant-based and high-fiber diets are also associated with increases in the Bacteroidetes phylum [160,162], or the Bacteroidetes/Firmicutes ratio, as well as microbial richness/diversity [142,155,160,162,163,164], in contrast to diets high in fat [165,166,167,168,169]. This is relevant in that various microbes from the Bacteroidetes phylum encode carbohydrate-active enzymes (CAZymes) necessary for degrading indigestible carbohydrates [139], and the Bacteroidetes:Firmicutes ratio may have implications for obesity and metabolic diseases, although the relationship is not clear as findings have been inconsistent [170,171].

Differences in gut microbial composition are not always observed in cross-sectional studies comparing vegans or other vegetarians with non-vegetarians [172]. In the AHS-2 cohort, only subtle differences were noted in the microbiome [173]. However, vast differences were discovered in the plasma metabolome, with vegans showing higher abundance of anti-inflammatory plant/polyphenol or microbial-related metabolites [174]. Non-vegetarians on the other hand may have higher abundance of amino acids and lipids conceivably associated with cardiometabolic phenotypes [174,175,176]. Intestinal microbiota convert choline and L-carnitine, derived from meat, fish, dairy, and eggs, into trimethylamine, which is oxidized by the liver to trimethylamine N-oxide (TMAO), a pro-inflammatory compound that has been associated with increased cardiometabolic risk [177,178,179,180]. Thus, it may be that microbial function is more relevant than composition, with metabolic profiles showing much greater differences, reflecting phenotypic changes.

There are physiological consequences of diet-induced shifts in the microbiome. Low consumption of plant-based foods may lead to increased penetration of the intestinal barrier, as a low-fiber diet triggers a shift from fiber-degrading to mucus-degrading bacteria [181]. This in turn could promote a hyperactive immune response, conceivably with the production of pro-inflammatory metabolites that fuel disease processes [182]. However, much remains to be understood about how vegetarian and plant-based dietary patterns impact the microbiome and associated metabolic responses to influence disease processes.

## 7. Plant-Based Diets and the Life Cycle

Vegetarian, including vegan, diets can satisfy the nutritional requirements of all stages of the life cycle. They can promote normal growth and development in infancy, childhood, and adolescence and meet the needs for energy and nutrients of these life cycle stages as well as those of pregnancy, lactation, and older adulthood.

### 7.1. Pregnancy and Lactation

Vegetarian diets can effectively meet energy and nutrient needs in pregnancy and lactation [183,184]. Several reviews, while noting the limited amount of information about vegetarian, including vegan, diets in pregnancy, have concluded that, with adequate nutrient intake, these diets are safe in pregnancy [183,185]. When food access is satisfactory, infant birth weights and the duration of gestation are similar in vegetarian and nonvegetarian pregnancy [186,187]. Some studies report that vegetarians are more likely to have infants who are small for their gestational age [188,189,190]. This finding may be due to lower mean pre-pregnancy BMI, lower weight gain, or inadequate weight gain in pregnancy. Well-nourished vegetarians produce nutritionally adequate breast milk that supports infant growth and development [191].

Health benefits of vegetarian diets in pregnancy include a lower risk of excessive weight gain and higher fiber and folate intakes [188,192,193]. Dietary patterns that are high in plant foods are associated with a reduced risk of gestational diabetes mellitus, hypertensive disorders of pregnancy, and preterm birth [194].

Nutrient requirements in vegetarian pregnancy and lactation generally do not differ from those for nonvegetarians [195]. Vegetarians may especially benefit from guidance on sources of iron, zinc, vitamin B12, iodine, and docosahexaenoic acid (DHA). Although iron absorption increases in pregnancy [196], iron needs are high, so iron-rich foods and low-dose iron supplements are recommended for all women [197,198]. The increased need for zinc can be met through a combination of increased intake and absorption [199]. Phytate’s inhibitory effect on zinc absorption is markedly reduced in late pregnancy and early lactation [200]. In addition to the use of iodized salt, a 150 μg/d iodine supplement is recommended for all pregnant and lactating women [201].

During pregnancy, blood DHA concentrations are often lower in vegetarians than in nonvegetarians [202]; cord blood DHA is lower in infants of vegetarians [202]. Breast milk DHA concentrations of vegetarians and vegans are lower than worldwide averages [203]. DHA or omega-3 supplementation is associated with greater gestational duration and a reduced risk of preterm birth [204,205]. Supplemental DHA derived from microalgae should be used in vegetarian pregnancy and lactation [195].

Adequate vitamin B12 intake is especially important during periods of growth such as pregnancy and breastfeeding. Infants born to long-term vegan mothers and who are breastfed are at risk of B12 deficiency. This is especially true when the mother’s diet is not well-supplemented. Symptoms of B12 deficiency in breastfed infants and small children fed a vegan diet include developmental delay or psychomotor regression, lethargy, anemia, neurological issues, and failure to thrive [206]. Pregnant and lactating vegetarians should consume reliable sources of vitamin B12, such as supplements and/or fortified foods, on a daily basis [195].

### 7.2. Infants, Children, and Adolescents

Vegetarian, including vegan, diets that are nutritionally adequate are appropriate for use in infancy, childhood, and adolescence and support normal growth [184,207,208]. Health benefits of vegetarian diets in childhood and adolescence include the potential for exposure to a wide variety of plant foods, lower risk for childhood obesity [209], and higher consumption of fruits and vegetables [210,211]. Vegan children appear to have lower intakes of total and saturated fat and cholesterol compared to non-vegan children [211]. A low-fat vegan diet has effectively treated children with obesity and elevated blood pressure [212].

Exclusive breastfeeding is recommended for infants for the first 6 months after birth, with breastfeeding continuing until at least 12 months of age [213]. If breastfeeding or exclusive breastfeeding is not possible, commercial infant formula should be used as the primary beverage for the first year. Plant milks, unmodified cow’s milk, other milks, and homemade formulas should not be used to replace breast milk or formula during the first year. Standard practices should be used when introducing complementary foods to vegetarian infants. Vegetable proteins, such as pureed beans or tofu, are used in place of pureed meats. After the first year, if toddlers are growing normally and eating a variety of foods, fortified soy or pea protein milk or dairy milk can be started [195].

Several nutrients require special attention in the planning of nutritionally adequate diets for young vegetarians, including iron, zinc, iodine, and vitamin B12; calcium and vitamin D may also require attention, depending on dietary choices and other factors. Protein recommendations for vegan children may be somewhat higher than standard recommendations because of factors including protein digestibility and amino acid composition [195]. Protein needs of vegetarian or vegan children and adolescents are generally met when their diets contain adequate energy and a variety of plant protein sources. Deficiencies of iron and zinc are rarely seen in vegetarian children eating varied diets [207]. Zinc supplementation may be needed when complementary foods are introduced, if foods are mainly those with low zinc bioavailability [214]. Iron and zinc status in infants, children, and adolescents should be monitored, and fortified foods and/or supplements used as needed. Iodized salt is a reliable source of iodine for children and teens. If maternal vitamin B12 intake or status are inadequate, breastfed infants should be given supplemental vitamin B12 [206]. Vegetarian children and adolescents should use vitamin B12-fortified foods or supplements to supply adequate vitamin B12. Calcium sources for children and adolescents include fortified plant-based milks, green leafy vegetables, and dairy products.

### 7.3. Older Adults

Older adults generally have decreased energy requirements, although nutrient requirements are often similar to, or higher than, those of younger adults. The selection of nutrient-dense diets is especially important for older adults. Limited research indicates that nutrient intakes of older vegetarians are comparable to those of older non-vegetarians [195].

Recommendations for calcium, vitamin B6, and vitamin D are higher for older adults [215,216]. There is some evidence that protein needs increase as well [217]. Higher protein foods such as soy products (including tofu, soy beverage, soy yogurt alternative, etc.), legumes, nuts and seeds, and meat analogs should be used two to three times a day by older vegetarians. Vitamin B6 recommendations for all older adults are higher due to decreased absorption and alterations in metabolism [216]. Vegetarians generally have adequate intakes of vitamin B6. Sources include potatoes, bananas, fortified breakfast cereals, and spinach. Several factors increase older adults’ risk for vitamin D insufficiency, including reduced dermal and renal synthesis [218,219], inadequate dietary intake, and limited sun exposure. Fortified foods and/or supplements may be needed for older adults to meet recommendations for calcium and vitamin D.

The main cause of vitamin B12 deficiency in older adults is impaired absorption of vitamin B12 from foods [220]. Absorption of purified vitamin B12 from fortified foods and supplements is not typically impaired, so recommendations call for older adults to use fortified foods and supplements as their primary sources of vitamin B12 [216].

## 8. Athletic Performance

Vegetarian diets can meet the needs of athletes at all levels, from recreational to elite athletes [221,222], and have been followed by athletes throughout history [223]. While a nutritionally adequate plant-based diet is thought to help optimize training and performance, due in part to its high carbohydrate [223,224,225] and high phytochemical content [225], limited evidence from well-controlled studies suggests that vegetarian diets neither enhance nor impair performance [225]. Additional research is needed to determine whether such diets enhance recovery and attenuate the oxidative damage and inflammation that occur with heavy training.

Nutrition recommendations for athletes should consider each athlete’s training volume (intensity and frequency), sport, season, performance goals, and food preferences. Vegetarian diets that meet energy needs and contain a variety of plant-based protein sources, including soy foods, dried legumes, nuts, seeds, quinoa, and other grains, can provide adequate protein to support most training needs. There is some evidence that plant-derived proteins result in lower post-prandial muscle protein synthesis responses compared with equivalent amounts of animal-derived proteins [226]; this response may be improved by consuming blends of different plant-derived proteins [226]. Milk and eggs [227,228,229] can supplement plant-based sources for vegetarian athletes.

Depending on food preferences, athletes need to ensure they consume adequate amounts of the nutrients that are either found less abundantly in vegetarian foods or are less well absorbed from plants compared to animal sources. These nutrients include calcium, iron, zinc, iodine and vitamin B12. For example, female athletes and endurance athletes should ensure sufficient consumption of iron-rich plant foods along with dietary factors that enhance rather than inhibit iron absorption [230,231,232]. Female athletes with restricted intake and amenorrhea (i.e., low energy availability) [233] may require additional calcium (1500 mg/day along with 1500–2000 IU vitamin D) to optimize bone health [234]. Maintaining adequate vitamin D status is important for athletes due to its role in immune function, inflammatory modulation, physical performance, and overall health [235,236,237,238]. Vegetarian athletes may have lower blood and muscle creatinine and carnitine concentrations [239,240,241,242] compared to omnivores due to lower dietary intake. Athletes participating in resistance training and bouts of high-intensity exercise may benefit from creatine supplementation [243], but there is no recognized benefit to carnitine supplementation. Vegetarian athletes, like most others, may benefit from education about food choices to optimize health and peak performance [244].

## 9. Nutrients of Concern in a Plant-Based Diet

### 9.1. Calcium

In addition to its role in bone mineralization, calcium is required for blood clotting, muscle function, nerve transmission, hormone release, intracellular signaling, and regulating key enzymes [245]. Typically, vegans consume substantially less calcium than other vegetarians and omnivores [192,246]. When calcium intakes are low, the body can compensate somewhat by increasing the fractional calcium absorption [247] and decreasing urinary calcium excretion [215]. However, anyone, including all types of vegetarians, with inadequate calcium intake needs to consistently use calcium-fortified foods, such as fortified breakfast cereals, fortified fruit juices, and fortified plant-based beverages, or take a calcium supplement, to meet their calcium needs. Vegan diets in the UK have been associated with a clinically significant increased risk of fracture when the calcium intake was inadequate [248].

Phytic and oxalic acids in plant foods are both inhibitors of calcium absorption. The calcium absorption from oxalate-rich vegetables (spinach, Swiss chard) may be as low 5%; from beans, almonds, tahini, and figs 20–25%; from dairy products 32%; from soy products (tofu, fortified soy beverages), it is similar to dairy milk; and from low-oxalate vegetables (kale, Chinese cabbage, broccoli, bok choy, etc.) 50–60% [249,250,251]. Boiling can reduce oxalate content in green leafy vegetables [252]. A vegetarian diet, with its high intake of fruit and vegetables, is rich in anti-inflammatory phytonutrients, specifically carotenoids and flavonoids, and potassium and magnesium. Carotenoids and flavonoids are associated with an improved BMD and lower bone fractures [253,254,255,256].

Compared to a vegetarian diet, consuming an animal protein diet is associated with an increased loss of urinary calcium [257].

### 9.2. Iron

In addition to its ability to transfer oxygen by means of hemoglobin and myoglobin, iron functions as a co-factor for many important enzymes (such as myeloperoxidase, important for immune function) and has a role in thyroid hormone synthesis and amino acid metabolism [245]. Since heme iron is generally better absorbed (15–30%) than non-heme iron (typically 5–10%), omnivores are assumed to have better iron status. However, vegetarians who eat a varied and well-balanced diet do not appear to be at any greater risk of iron-deficiency anemia than omnivores [258,259]. Hemoglobin levels of the two dietary groups normally show no significant differences [259,260]. Additional studies of iron deficiency in vegetarians are needed before definitive conclusions can be reached. A varied diet that is rich in wholegrains, legumes, nuts, seeds, dried fruits, iron-fortified cereal products, and green leafy vegetables provides an adequate iron intake. Vegetarian diets generally contain as much or more iron than omnivore diets [92,195].

Non-heme iron absorption is significantly affected by several dietary components [261,262]. Vitamin C, other organic acids (citric, malic, lactic, tartaric acids), and erythorbic acid (an antioxidant used in processed food) all enhance absorption [196,230,259,263,264]. Plant ferritin, found in soy and other legumes, is an easily absorbed source of iron (22–34%). While phytates (found in legumes, nuts, and whole grains) can inhibit non-heme iron absorption, their inhibitory effect is diminished by baking, soaking, leavening, and germination [184,265]. Furthermore, the overall long-term effect of enhancers and inhibitors of iron may be less important than once thought when the foods are eaten as part of a whole diet [266,267].

Absorption of non-heme iron is also inversely related to the body’s iron status [196]. When stores are low and the need for iron increases, compensatory mechanisms facilitate greater absorption of iron. Absorption can be as low as 2–3% in people with good iron stores but as high as 14–23% in people with low iron stores [268].

Humans have a limited ability to excrete excess stored iron [258], so consuming large amounts of heme iron may be unhealthy due to its pro-oxidant nature. Consumption of heme iron has been associated with an increased risk of chronic diseases such as diabetes, metabolic syndrome, and colorectal cancer [269,270,271,272]. Vegetarians typically have lower iron stores (as reflected in lower serum ferritin levels), which may be an advantage as lower serum ferritin levels may be associated with improved insulin sensitivity and reduced risk of T2D [258,273].

Iron absorption from an omnivorous diet is claimed to be about 18%, whereas for a plant-based diet it is said to be about 10% [196]. Hence, the current Dietary Reference Intake (DRI) for iron for vegetarians has been set 1.8 times higher than that for non-vegetarians. This increased requirement is based on limited research, which has been unable to accurately measure adaptive absorption rates of non-heme iron in vegetarians [267,274]. Further research is needed to reassess the iron requirement recommended for vegetarians [232].

### 9.3. Zinc

Zinc acts as a coenzyme for multiple enzymes involved with growth, immunity, cognitive function, bone function, and regulation of gene expression [275,276]. Zinc deficiency causes stunted growth, poor appetite, dermatitis, alopecia, endocrine dysfunction, and impaired immunity [276]. Zinc deficiency is not any more commonly seen in vegetarians than in non-vegetarians [277]. Zinc intake and serum levels for adolescent and adult vegetarians in developed countries are the same or slightly lower than for omnivores, but within the normal range [214,231,275,278,279]. In developing countries, vegans and vegetarians are more likely to show marginal zinc status [278].

The bioavailability of zinc from plant foods may be reduced. However, zinc absorption and retention can be regulated by homeostatic mechanisms, adapting to lower intakes by reducing losses and increasing absorption [275]. During periods of high demand (pregnancy, infancy), absorption becomes more efficient [280].

Phytates in cereals and legumes lower absorption of zinc, but leavening, soaking, fermenting, or sprouting reduces the phytate levels and makes zinc more bioavailable [281]. Sulfur-containing amino acids and organic acids in a variety of plant foods will also enhance zinc absorption [279,282].

Vegetarian food sources for zinc include nuts, seeds, wholegrains, legumes, tofu, tempeh, and dairy products [283]. The use of supplements and fortified foods (such as fortified breakfast cereals) may be necessary for very restricted vegan diets [214,246].

### 9.4. Iodine

Iodine is essential for thyroid hormones, which regulate metabolic activity. Iodine is especially important in pregnancy for fetal development and during early childhood. Iodine deficiency in childhood can prevent children from attaining their full physical potential and intellectual capacity [284].

Major dietary sources of iodine include iodized salt, seafood, and dairy products [284]. The iodine content of seaweeds and dairy products can vary widely [195,285]. Sea salt, Himalayan salt, and the salt used in processed foods typically do not contain iodine [195]. Although foods such as soybeans, cruciferous vegetables, and sweet potatoes contain natural substances that interfere with iodine uptake by the thyroid, these foods have not been associated with thyroid dysfunction in healthy people, provided iodine intake is adequate [196,286].

Vegans who do not use iodized salt and/or sea vegetables may have low iodine intakes and may be at risk for iodine deficiency [287,288].

### 9.5. Vitamin B12

Vitamin B12 is required for red blood cell formation, DNA synthesis, homocysteine metabolism, and the myelination and function of the central nervous system [289]. Vitamin B12 deficiency is not uncommon among the elderly and unsupplemented vegans. It can manifest itself with consequential hematological and neurological changes. Typically, the mean dietary intake of vitamin B12 of vegans falls well below the DRI, while that of lacto-ovo-vegetarians may be marginal, depending on the use of dairy products [246,290,291]. Vegans must obtain their vitamin B12 either from regular use of vitamin B12-fortified foods, such as fortified plant-based beverages, fortified breakfast cereals, fortified vegetarian meat analogs, or from a regular vitamin B12 supplement. Unfortified plant foods such as fermented soy foods, leafy vegetables, seaweeds, mushrooms, and algae (including spirulina) do not contain significant amounts of active vitamin B12 to provide daily needs [292]. Furthermore, a number of medications can impair the absorption or utilization of B12. Vitamin B12 appears to be a cofactor involved in the production of nitric oxide [293], which would have important implications for vascular and immune health.

About 50% of dietary B12 is normally absorbed via ileal receptors, mediated by the intrinsic factor, a glycoprotein from the stomach. The ileal receptors become saturated with 1.5 to 2 µg of B12, limiting further absorption [216]. When ingesting large doses of supplemental B12, about 1% of the dose is absorbed by passive diffusion across the intestinal tract [216]. Daily needs can be adequately met in non-pregnant, non-lactating people by consuming a 500 µg B12 supplement at least three times a week. Vitamin B12 is well absorbed from either sub-lingual or chewable tablets. While the methylcobalamin supplement is touted as the more effective form of B12, its bioavailability is not superior to that of cyanocobalamin, which is the more stable and most commonly used form of B12 in fortified foods and many supplements [294,295].

A deficiency of vitamin B12 may take years to develop in adults, as most of the B12 secreted into the gut via the bile gets reabsorbed, thus conserving the body stores [216]. Therefore, a regular consumption of adequate B12 is important to avoid a sub-clinical deficiency that can go undetected for years. An elevated serum methylmalonic acid (MMA) level is a reliable indicator of B12 deficiency [216], while the serum B12 level is an insensitive indicator of B12 status. While serum B12 levels between 148 and 221 pmol/L (200–300 pg/mL) are considered borderline B12 deficiency [296], some individuals with B12 values in this range manifest neuropsychiatric problems and memory loss [297]. As a good preventative measure, all vegans should annually check their B12 status.

### 9.6. Vitamin D

Vitamin D facilitates calcium absorption from the gut, regulates bone mineralization, cell growth, and differentiation. Its other roles include reduction of inflammation and modulation of neuromuscular and immune function [298]. Because cutaneous production of vitamin D from sunlight exposure is not adequate (especially in the elderly, dark-skinned individuals, and heavy sunscreen users) to meet nutrition needs in populations living in high latitudes, especially during the winter months, regular food and supplement sources are necessary. Foods contain limited amounts of vitamin D, so supplements are often needed to meet needs. Depending upon one’s age, geographical location, dietary preferences, and body weight, a daily supplemental dose of 10–50 µg (400 to 2000 IU) of vitamin D may be needed to achieve optimal serum levels of 25-hydroxyvitamin D (25(OH)D) year-round [299].

One study found no significant difference in serum 25(OH)D levels between vegetarians and non-vegetarians. Factors such as vitamin D supplementation, degree of skin pigmentation, and amount of sun exposure had a greater influence on serum 25(OH)D levels than did diet [300]. By contrast, in the large EPIC-Oxford study, plasma 25(OH)D levels in British vegetarians were 14.3% lower, and in vegans 27.5% lower, than in meat eaters [301].

Vitamin D intake by vegans tends to be substantially below that of lacto-ovo-vegetarians and omnivores [195]. Low serum 25(OH) D levels and reduced bone mass have been reported in vegans living in high latitudes who were not using vitamin D-fortified foods or supplements [302,303].

Fortified plant-based beverages, fortified orange juice, ready-to-eat breakfast cereals, and fortified margarines provide vitamin D for vegetarians. Modest levels of vitamin D are also obtained from mushrooms that have been exposed to ultraviolet light under controlled conditions [304]. Lacto-ovo-vegetarians also obtain vitamin D from fortified dairy products and eggs. Depending on sunlight exposure and dietary intake, supplements may be needed. For low daily doses, vitamin D2 appears to be as effective as vitamin D3 in maintaining circulating levels of serum 25(OH) D [305]. When given as a single large dose, vitamin D2 appears to be less effective than vitamin D3 for improving the vitamin D status [306].

With appropriate food and supplement choices, a vegetarian diet can be consistent with having an adequate vitamin D status and supporting a healthy BMD (bone mineral density) [129].

### 9.7. Omega-3 Fatty Acids

Omega-3 fatty acids (n-3) are associated with favorable cardiometabolic status [307]. The source of omega-3 for vegetarians is predominantly α-linolenic acid (ALA) [308]. Normally, only small amounts of ALA are converted to the longer-chain eicosapentaenoic acid (EPA), and to a less degree DHA, particularly if linoleic acid intake is high [308,309]. Conversion of ALA is also affected by health status, age, dietary composition, and gender [310]. Results from the EPIC-Norfolk cohort study revealed that omega-3 status differences were much smaller than dietary differences, with vegans and vegetarians showing a more efficient conversion of ALA to EPA and DHA [311]. Most studies indicate that plasma, serum, erythrocytes, adipose, and platelet levels of EPA and DHA are lower in vegetarians than omnivores [309], yet there is no evidence of adverse effects on heart health or cognitive function in vegetarians [312,313].

EPA has antithrombotic properties and confers cardiovascular protection [308,314], while DHA has been linked to eye and brain development and is important for ongoing visual, cognitive, cardiovascular health [308,315]. Omega-3 fatty acids may also help regulate gut microbiota and immunity and reduce the risk of inflammatory diseases [316,317,318]. ALA, EPA, and DHA intakes are all associated with a reduced risk of CVD [319].

The richest sources of ALA include flaxseed, hemp seed, walnuts, chia seeds, and their oils, with smaller amounts present in canola and soy oils, and green leafy vegetables [310]. Currently the National Academy of Medicine (Washington, DC, USA) has not established recommendations for EPA and DHA, while the European Food Safety Authority has recommended an intake of 250 mg/day for EPA and DHA [320]. To date, an adequate intake of ALA has been specified as 1.6 g for men and 1.1 g for women [321]. The ideal omega-6/omega-3 ratio for optimal health has not been defined, although various authors have debated the issue [321]. Improving the DHA status of an individual is generally regarded as desirable. For the vegetarian, a regular use of an algal DHA supplement would be an effective way to increase serum DHA levels [309,322].

The critical period of pregnancy and lactation requires a higher n-3 status (particularly DHA) [308,323,324]. Pregnant and breastfeeding women, and those at greater risk for poor ALA conversion, such as people with diabetes, older people, and premature infants, are most likely to benefit from DHA supplements derived from micro-algae [319,325]. Omega-3-rich eggs and DHA-fortified foods are also food sources of DHA for vegetarians.

### 9.8. Protein

Individuals following vegetarian diets generally consume more than adequate protein, particularly in western countries, although intakes are typically lower than those of omnivores [120]. Furthermore, as long as a variety of protein-rich foods are consumed, vegetarian diets are able to provide all of the indispensable amino acids [120,326]. While there is no need for different protein foods to be combined in one meal, a variety of plant foods should be included each day [326]. Most plant foods contain some protein, with the best sources being legumes, soy foods (including fortified soy milk, tofu, and tempeh), nuts, and seeds. Grains and vegetables also contain protein but in smaller amounts.

While the lower protein intake and quality of protein in a vegetarian diet is often cited as a concern, there is increasing evidence for the health benefits of consuming protein from plant sources rather than animal sources, and this may be one of the reasons why vegetarians have a lower risk of obesity and chronic diseases [327].

Those consuming omnivorous diets in western countries tend to get 1.5 to 2 times the recommended protein intake, and such high protein intakes can have a variety of deleterious effects, such as increased calcium excretion and reduced insulin sensitivity [328,329].

## 10. Guidelines for Health Professionals

Significant health benefits are associated with vegetarian, including vegan, diets. Plant-based diets, even if not completely vegetarian, also offer significant health benefits. Health professionals should discuss the benefits of vegetarian and near-vegetarian diets with their clients and provide supportive, reliable, evidence-based information and resources. If the practitioner is unfamiliar with vegetarian nutrition, clients should be referred to other health professionals with expertise in this area, such as registered dietitians.

Health professionals are ethically obligated to respect vegetarian dietary patterns and to provide information so that clients are aware of their nutritional needs, sources of nutrients, and any dietary modifications needed to meet their individual situation. The client’s food preferences should be determined and respected. This may include religious or cultural factors that influence one’s food choices.

Health professionals who work with vegetarians and those interested in vegetarian diets should be familiar with current research on vegetarian nutrition as well as with vegetarian foods and food preparation. There are a number of excellent books and other resources available for health professionals to acquaint themselves with evidence-based data [195,222,330,331]. Individualized counseling materials should be developed that feature vegetarian foods.

Some traditional cultures have plant-based traditions. When working with clients from these cultures, professionals should focus on the retention of healthy traditional practices, with modification of other practices to promote more healthful diets instead of promoting the eating patterns of the dominant culture [332].

It is incumbent on any health professional providing counsel regarding dietary choices to remember it is not what a diet is called, but what foods an individual consumes on a regular basis that determines the adequacy of a diet.

## 11. Conclusions

Plant-based diets continue to grow in popularity. Currently, there is a vibrant interest in the sustainability of diets and a growing awareness of the need to focus on both human health and the health of the planet in formulating dietary guidelines. Plant-based diets are more sustainable than diets based on animal products, since they use fewer natural resources and produce fewer GHG emissions. Vegetarian and vegan diets provide protection against a number of common chronic diseases, such as CVD, obesity, T2D, and certain types of cancer. The consumption of a plant-based diet rich in fiber and phytochemicals not only provides disease-preventing benefits but also has a substantial impact on the composition and function of the gut microbiome, which in turn influences overall health.

Both a vegetarian and vegan diet are appropriate for all stages of the life cycle, including pregnancy and lactation, all stages of childhood, the elderly, and for athletes. When appropriately planned, a plant-based diet (consisting substantially of minimally processed foods) can be nutritionally adequate. Vegetarians and especially vegans should consume a well-balanced diet and regularly use fortified foods and/or supplements. Special attention should be paid to calcium, iron, vitamin D, and vitamin B12. A deficiency may be exacerbated when supplements are not utilized and when food choices are limited or self-restricting.

Health professionals who work with vegetarians and those interested in vegetarian diets should be familiar with current research on vegetarian nutrition and be able to provide information so that clients are aware of their nutritional needs, sources of nutrients, and any dietary modifications needed to meet their individual situation. The health professional should be sensitive to the client’s food preferences and respect any religious or cultural factors that influence their food choices.

## Data Availability

Not applicable.
